# Kinetics of Nirogacestat-Mediated Increases in B-cell Maturation Antigen on Plasma Cells Inform Therapeutic Combinations in Multiple Myeloma

**DOI:** 10.1158/2767-9764.CRC-24-0075

**Published:** 2024-12-11

**Authors:** Todd Shearer, Melissa Comstock, Rex L. Williams, Mark C. Johnson, Ewa Cendrowicz, Cathrine Leonowens, Margaret Smith, Todd M. Baughman, Caroline J. Breitbach, Shinta Cheng, Damian J. Green

**Affiliations:** 1 SpringWorks Therapeutics, Inc., Stamford, Connecticut.; 2 Fred Hutchinson Cancer Center, Translational Science and Therapeutics Division, Seattle, Washington.; 3 ICON BioAnalytical Laboratories, Assen, the Netherlands.; 4 Independent Consultant, Sanford, North Carolina.; 5 Division of Transplantation and Cellular Therapy, Department of Medicine, Sylvester Comprehensive Cancer Center, University of Miami, Miami, Florida.

## Abstract

B-cell maturation antigen (BCMA) is the target of several investigational and approved drugs for multiple myeloma. BCMA expressed on plasma cells (PC) and multiple myeloma cells is cleaved by the enzyme γ-secretase, reducing membrane-bound BCMA (mbBCMA) receptor density. γ-Secretase inhibitors (GSI) have been shown to increase mbBCMA density and may enhance efficacy of BCMA-targeted therapies. The pharmacodynamic profile of the GSI nirogacestat was evaluated in multiple myeloma cell lines and a phase I study in healthy volunteers. In multiple myeloma cell lines, mbBCMA density and soluble BCMA concentrations were measured before and after short-duration nirogacestat exposure and at serial time points following washout. In the phase I study, 23 participants were administered a single oral dose of nirogacestat 50, 150, or 300 mg or repeated doses of 100 mg every 12 hours for up to 48 hours; mbBCMA density on PCs (from whole blood and bone marrow) and nirogacestat plasma concentrations were measured at baseline and postdose. After single-dose administration, serum nirogacestat concentrations rapidly increased (*T*_max_ ∼1 hour), and a two-compartment model with linear absorption and clearance best described nirogacestat pharmacokinetics. In multiple myeloma cells and healthy volunteers’ PCs, nirogacestat resulted in rapid and robust increases in mbBCMA density, with increases up to 20-fold within 4 to 8 hours of exposure. Concomitant decreases in soluble BCMA were observed. Nirogacestat is currently being evaluated in combination with several BCMA-directed therapeutic agents in patients with multiple myeloma. Elucidating the kinetics of BCMA in response to nirogacestat is key to guiding dosing and therapeutic strategies in multiple myeloma.

**Significance:**

GSIs can enhance multiple myeloma therapies targeting BCMA by increasing mbBCMA on plasma cells. In response to the GSI nirogacestat, mbBCMA rapidly and robustly increased *in vitro* and *in vivo*. Elucidating nirogacestat’s effects on BCMA kinetics will guide potential multiple myeloma dosing strategies.

## Introduction

Multiple myeloma, a cancer of plasma cells (PC), constitutes ∼10% of hematologic malignancies in the United States (1, 2). Although treatment options have improved considerably in the last decade, with therapies such as proteasome inhibitors, immunomodulatory drugs, and mAbs becoming available, the 5-year survival rate for patients with multiple myeloma is ∼50% and can be lower in those with high-risk cytogenetic characteristics (2, 3). The disease remains generally incurable, and patients usually require multiple rounds of therapy to manage relapsed disease that has become refractory to prior lines of treatment (2–4). Combining different classes of drugs has increased the frequency and depth of response, but the need for expanded treatment options is critical (2–4).

B-cell maturation antigen (BCMA), a receptor expressed on the membrane of normal PCs and multiple myeloma cells, is essential for the survival of long-lived bone marrow PCs (3, 5, 6). BCMA is the target of several investigational agents and approved products for the treatment of multiple myeloma because of expression that is largely restricted to aberrant and normal PCs (2, 3). Mechanistically, BCMA is shed from the cell surface by the intramembrane cleaving enzyme, γ-secretase, resulting in reduced density of membrane-bound BCMA (mbBCMA) and release of soluble BCMA (sBCMA; ref. 7). Low mbBCMA density or loss of mbBCMA expression may impair the rate and durability of response and confer resistance to BCMA-targeted therapies (8–11).

In multiple myeloma treatment, γ-secretase inhibitors (GSI) are being explored as adjunctive therapy to optimize the effects of BCMA-targeted therapies by increasing mbBCMA density (9, 10, 12–14). Although the effect of GSIs on mbBCMA density has been well characterized *in vitro*, their effect on BCMA dynamics in humans is not well understood. An early barrier to understanding BCMA dynamics was the inability to assess treatment-related changes in mbBCMA in paraffin-embedded samples with IHC. This barrier has been overcome with quantitative flow cytometric methods on fresh samples (15). Recently, effects on BCMA dynamics were reported from a first-in-human phase I study evaluating GSI use in combination with a BCMA-targeted therapy in patients with multiple myeloma (*n* = 18 treated; ref. 10). Substantial increases in mbBCMA density on malignant PCs, decreases in sBCMA, and robust clinical responses were observed. In the setting of multiple myeloma, opportunities to evaluate the discrete impact of a GSI on BCMA dynamics are hampered by the fact that GSIs alone do not have antimyeloma tumor effects in multiple myeloma. Therefore, GSIs must be administered in combination with other multiple myeloma targeting interventions, thereby confounding the ability to understand the impact of the GSI alone. Because BCMA is both expressed on and shed from normal and malignant PCs through γ-secretase cleavage, PCs from healthy individuals provide a reasonable surrogate for assessing BCMA kinetics in response to GSI exposure and allow for independent elucidation of GSI treatment effects on BCMA dynamics.

Nirogacestat (OGSIVEO, SpringWorks Therapeutics, Inc. https://www.ogsiveo.com/) is an oral small-molecule, selective GSI approved for the treatment of adults with progressing desmoid tumors who require systemic treatment (16–18). Nirogacestat is also a candidate for potentiation of BCMA-targeted therapies for multiple myeloma. Clinical trials evaluating nirogacestat in combination with several BCMA-directed modalities, including antibody–drug conjugates and bispecific antibodies, are ongoing or planned (NCT05556798, NCT04093596, NCT05573802, NCT05259839, NCT05090566, NCT04126200, NCT04722146, and NCT05137054), and preliminary evidence is consistent with the hypothesis that nirogacestat can potentiate the efficacy of BCMA-directed treatment (19, 20). The various BCMA-targeting therapeutics have distinct affinities for BCMA and different timing for onset and duration of peak antitumor activity relative to dosing time. To inform decision-making about nirogacestat dose administration when paired with each BCMA-targeted therapy, it is critical to determine the impact of nirogacestat on absolute BCMA target antigen density and to quantify the duration of its effect.

We sought to characterize the pharmacodynamic (PD) effects of nirogacestat on BCMA over time using multiple myeloma cell lines and primary samples from a phase I dose-range study in healthy volunteers. The study in healthy volunteers allowed for the investigation of nirogacestat effects without the potential confounders of prior or concurrent treatment with BCMA-targeted therapies. The clinical study also evaluated PCs from bone marrow and whole blood to assess concordance and whether whole-blood sampling can be used as a surrogate for bone marrow biopsies to monitor BCMA dynamics.

## Materials and Methods

### Effect of nirogacestat on BCMA dynamics in multiple myeloma cell lines

The concentration–response relationship of nirogacestat and BCMA (mbBCMA and sBCMA) was evaluated in six multiple myeloma cell lines: MM.1R (ATCC, Cat. # CRL-2975, RRID: CVCL_8794, gift from Steven Rosen, Northwestern University), H929 (ATCC, Cat. # CRL-9068, RRID: CVCL_1600), MOLP-8 [Deutsche Sammlung von Mikroorganismen und Zellkulturen GmbH (DSMZ), Cat. # ACC 569, RRID: CVCL_2124], KMS-12-BM (DSMZ, Cat. # ACC 551, RRID: CVCL_1334), L-363 (DSMZ, Cat. # ACC 49, RRID: CVCL_1357), and U266 (ATCC, Cat. # TIB-196, RRID: CVCL_0566). The cells were in culture for 37 days from thawing to the described experiment. The experiment was performed on February 11, 2021. Fred Hutchinson Cancer Center (FHCC) used Rapid Mycoplasma Detection Kit (PCR) and IDEXX BioAnalytics used PCR Profile to test for *Mycoplasma*. FHCC used Cell Line DNA Fingerprint—CODIS and short tandem repeat matching to authenticate cell lines. ATCC used short tandem repeat matching to authenticate cell lines (following amplification on PowerPlex 18D Kit from Promega, processing using the ABI Prism 3500xl Genetic Analyzer, and analysis using GeneMapper ID-X v1.2 software). Details of the handling of cell line samples are as follows:

- MM.1R: original tube labeled (MM1R, 9e6, December 1, 2010, N. Orgun) thawed August 22, 2017, and created stock with freeze date September 20, 2017. ATCC CRL-2975. Authenticated September 25, 2017, at FHCC and *Mycoplasma*-negative test done September 25, 2017, at FHCC and March 16, 2020, at IDEXX.
- NCI-H929: original tube labeled (H929, March 26, 2011, N. Orgun) thawed December 1, 2017, and created stock with freeze date December 12, 2017. From ATCC CRL-9068. Authenticated June 9, 2017, at ATCC and *Mycoplasma*-negative test done June 6, 2017, at FHCC and March 16, 2020, at IDEXX.
- MOLP-8: original tube from DSMZ ACC 569 (lot 11, vial date August 10, 2016) received October 25, 2017, thawed November 3, 2017, and created stock with freeze date November 13, 2017,. *Mycoplasma*-negative test done April 14, 2021, at IDEXX.
- KMS-12-BM: original tube from DSMZ ACC 551 (lot 9, vial date July 29, 2016) received October 25, 2017, thawed November 3, 2017, and created stock with freeze date November 24, 2017. *Mycoplasma*-negative test done April 14, 2021, at IDEXX.
- L-363: original tube from DSMZ ACC 49 (lot 15, vial date April 22, 2016) received October 25, 2017, thawed November 3, 2017, and created stock with Freeze date November 13, 2017. *Mycoplasma*-negative test done April 14, 2021, at IDEXX.
- U266: original tube labeled (U266, December 29, 2011, N. Orgun) thawed January 22, 2018, and created stock with freeze date February 8, 2018. From ATCC TIB-196. Authenticated July 3, 2017, at FHCC and *Mycoplasma*-negative test done June 6, 2017, at FHCC and March 16, 2020, at IDEXX.

On day −1 of the experiment, multiple myeloma cell lines were plated in 96-well tissue culture plates in triplicate, and nirogacestat was added at concentrations of 0.1 to 1,000 nmol/L. Control wells without nirogacestat were included in triplicate. On day 0, cells were pelleted, and the supernatant was removed for analysis of sBCMA. Cells were washed twice and stained with a PE-labeled monoclonal antibody against human BCMA (CD269, BioLegend, Cat. # 357504, RRID: AB_2561926). Antibody-binding capacity was measured using a BD Quantibrite kit, and all flow cytometric sample data were acquired using a BD Celesta Cell Analyzer (BD Biosciences) and analyzed using FlowJo Software (FlowJo, RRID: SCR_008520). sBCMA was measured by ELISA in duplicate. To evaluate the time course of nirogacestat response and washout, cells were incubated with 250 nmol/L nirogacestat for 3, 6, 24, and 48 hours, and mbBCMA density and sBCMA concentration were assessed as described earlier. Nirogacestat was then washed out by rinsing cells with media three times; mbBCMA density and sBCMA concentration were assessed at 3, 6, 24, and 48 hours after washout. To evaluate the effect of exposure to different GSIs, 250 nmol/L nirogacestat, 100 nmol/L crenigacestat, or 1,000-nmol/L RO4929097 was added to multiple myeloma cell lines. mbBCMA density was measured before treatment and after 24 hours, as described earlier.

### Effect of nirogacestat on BCMA dynamics in healthy volunteers

#### Study overview

The primary objective of the study was to evaluate the PD effect of nirogacestat in relation to BCMA. Secondary objectives were to evaluate pharmacokinetics (PK), safety, and tolerability of nirogacestat after single- and multiple-dose administration. An exploratory objective was to establish an assay to measure mbBCMA density on PCs from whole blood as a surrogate for bone marrow.

This study was approved by the independent Ethics Committee of the Foundation Beoordeling Ethiek Biomedisch Onderzoek (Evaluation of Ethics in Biomedical Research) before eligibility screening. The study was conducted in accordance with international ethical guidelines, including the Declaration of Helsinki, Council for International Organizations of Medical Sciences International Ethical Guidelines, and applicable International Conference on Harmonization Good Clinical Practice guidelines, laws, and regulations. All participants gave written informed consent. All authors had access to primary clinical trial data. This study is registered in the European Union Drug Regulating Authorities Clinical Trials Database (EudraCT 2022-000386-40; www.clinicaltrialsregister.eu).

#### Study design

**Figure 1:**
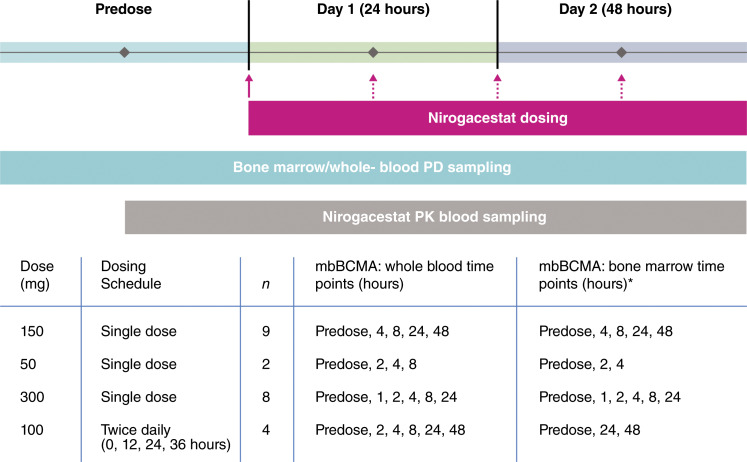
Nirogacestat dosing and sampling schedule. The solid arrow indicates nirogacestat single doses and the first dose of nirogacestat twice daily dosing; the dotted arrows indicate nirogacestat 100 mg twice daily doses. Baseline whole-blood and bone marrow samples were collected 1 day before dosing (predose). *Each dosed participant contributed one predose and one postdose bone marrow sample (*n* = 2 per posttreatment time point). Sampling times were based on the randomization/enrollment scheme shown. Rich PK sampling and all indicated whole-blood mbBCMA samples were collected from each participant through the assigned postdose time point.

This was an open-label, randomized, adaptive- and parallel-design, phase I study in healthy men (*n* = 23 treated with nirogacestat). The study was restricted to men because of clinical findings of ovarian toxicity in women of childbearing potential taking γ-secretase inhibitors (17). In part 1, fresh whole-blood and bone marrow samples (aspirates) were collected from untreated healthy participants (*n* = 3) for the development and validation of the mbBCMA density assay. In part 2, participants (*n* = 9) received a single 150 mg oral dose of nirogacestat. Matched whole-blood and bone marrow samples were collected before and after dosing, with sampling times assigned based on the randomization/enrollment scheme (Fig. 1). For bone marrow samples, each participant provided one predose and one postdose sample, with ≥2 participants assigned to each postdose time point (Fig. 1). The fold change in postdose mbBCMA density was evaluated by comparing the results with the participant-matched predose samples. The initial results from part 2 of the study were used to develop a pharmacokinetic–pharmacodynamic (PKPD) model predicting the PKPD relationship of nirogacestat and effects on mbBCMA density on PCs. Model simulations were utilized to help select doses and sampling times for part 3 of the study, which included oral administration of low (50 mg, *n* = 2) and high (300 mg, *n* = 8) single doses of nirogacestat or multiple doses of 100 mg nirogacestat every 12 hours for up to 2 days (*n* = 4). Matched whole-blood and bone marrow samples were collected as described earlier.

#### PK

Nirogacestat serum concentrations after single doses (50, 150, and 300 mg) and repeated doses (100 mg, twice daily)] were assessed through extensive PK sampling [blood samples taken predose (0 hour) and 0.5, 1, 1.5, 2, 3, 4, 5, 6, 8, 10, 12, 24, 36, and 48 hours postdose]. Serum concentrations of nirogacestat were determined using a validated LC/MS-MS assay.

#### PD and assay validation

To determine the PD effects of nirogacestat, the fluctuations in mbBCMA density on PCs from whole-blood and bone marrow aspirates were analyzed using flow cytometry (see details in the following paragraph). mbBCMA density was measured at baseline (predose) and 1, 2, 4, 8, 24, and 48 hours postdose; sampling times based on the dosing schedule and collected sample scheme are shown in Fig. 1. The assay was validated for reproducibility, stability, and sensitivity, with acceptance criteria of <30% coefficient of variance for reproducibility and <30% bias for stability assessments.

The antibody cocktail designed for the enrichment of PCs and analysis of mbBCMA included CD3-FITC, CD14-FITC, CD56-FITC, CD19-PerCP-Cy5.5, CD138-APC, CD38-BV421, and BCMA-PE. To facilitate gating of BCMA-positive cells, the same cocktail was used but with a PE-conjugated isotype control instead of BCMA-PE in a second tube. Whole-blood or bone marrow samples were mixed with an antibody cocktail, incubated for 15 minutes at room temperature, lysed/fixed for 15 minutes at room temperature, washed twice, and acquired on a flow cytometer. Samples were analyzed within 2 hours after collection.

Bone marrow aspirates were filtered through a 70-µm filter, and total white blood cell counts in matched whole-blood and bone marrow samples were enumerated in a Sysmex XS-1000i hematology analyzer. Samples were subsequently stained with an antibody cocktail designed for enrichment of PCs and analysis of mbBCMA, lysed-fixed, and acquired via a BD LSRFortessa X-20 Cell Analyzer cytometer equipped with BD FACSDiva Flow Cytometry Software (BD FACSDiva Software, RRID: SCR_001456).

Data were analyzed in FCS Express 6, Flow Clinical Edition Software (*De Novo* Software). A multiparameter gating scheme was used to isolate the rare PC population (Supplementary Fig. S1A) from whole-blood and bone marrow aspirates. Samples were first gated on live single-cell peripheral blood mononuclear cells followed by exclusion of non–B-cell populations using a dump channel composed of CD3, CD56, and CD14. The dump channel–negative population containing the PCs of interest was then gated on CD19 expression. PCs were isolated using CD38/CD138 gating before mbBCMA final analysis. The density of mbBCMA on PCs at baseline and after nirogacestat treatment is shown in Supplementary Fig. S1B. Molecules of equivalent soluble fluorochrome of mbBCMA were calculated using SPHERO Ultra Rainbow Calibration Particles Kit (Spherotech).

#### sBCMA measurements

The capture antibody was diluted to 1 μg/mL, and 50 μL/well was added to a Meso Scale Discovery (MSD) 96-well streptavidin plate. After an 1-hour incubation at room temperature, the plate was washed three times. Samples, standards, and controls were added to the wells (each at 50 μL/well), and after a 1-hour incubation, the plate was washed three times. Detection antibody diluted to 0.125 μg/mL was added to each well at 50 μL/well, and after a 1-hour incubation, the plate was washed three times. A total of 150 μL of MSD Read Buffer was added to each well, and the plate was read using an MSD S600 plate reader (Meso Scale Diagnostics). Pooled normal human serum was used as a quality control matrix: The baseline BCMA concentration was determined in 19 replicates in five runs (7,026 pg/mL).

#### Population PKPD model

A “fit-for-purpose” model was developed to describe the PKPD relationship between the PK of nirogacestat and the PD response (changes in BCMA density on PCs). All dataset preparation modeling was performed in R version 4.3.0. The following criteria were utilized during model development and selection: objective function value, condition number, precision (relative SE) and plausibility of parameter estimates, values of interindividual variability and residual error, standard goodness-of-fit plots, visual predictive checks (VPC), and prediction-corrected VPCs. Because of the limited dataset available for bone marrow, modeling was restricted to PCs isolated from whole blood. Correlation analysis was performed to evaluate whether the measurement of BCMA density on PCs in whole blood was a good surrogate for bone marrow.

#### Safety

Adverse events, including treatment-emergent adverse events (TEAE) and serious adverse events, were recorded throughout the study. The severity of adverse events was graded using the Common Terminology Criteria for Adverse Events, version 5.0, condition-specific scale.

### Data availability

Data were generated by SpringWorks Therapeutics and the Fred Hutchinson Cancer Center. The data generated in this study are not publicly available because of patient privacy requirements but are available upon reasonable request from the corresponding author following deidentification/anonymization in accordance with applicable law.

## Results

### Effect of nirogacestat on BCMA dynamics in multiple myeloma cell lines

**Figure 2:**
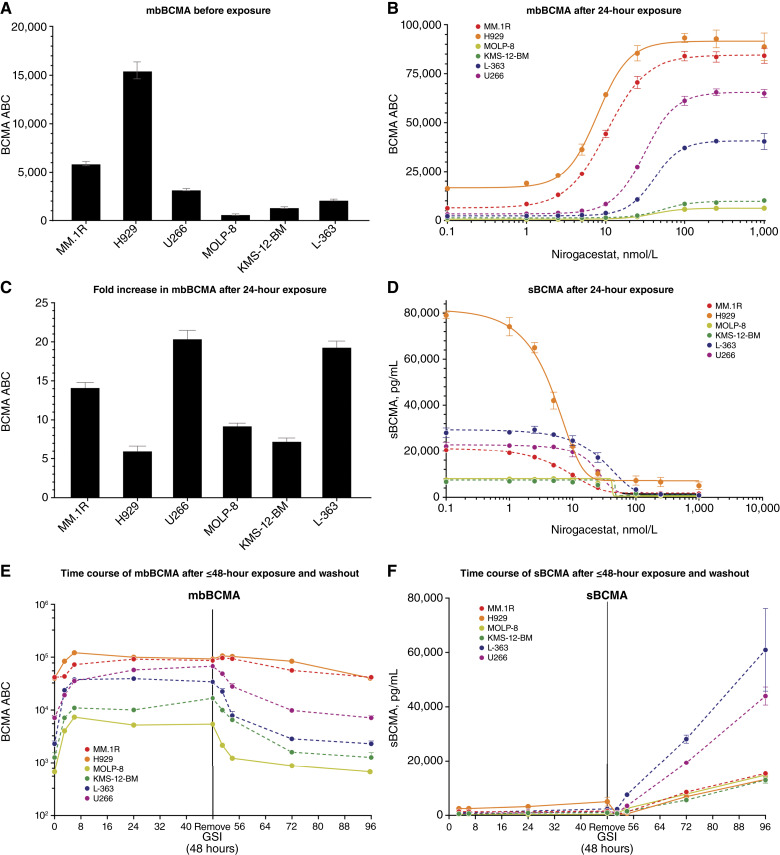
BCMA dynamics in multiple myeloma cells before and after nirogacestat exposure. mbBCMA density in six multiple myeloma cell lines was measured (**A**) before and (**B**) after 24-hour exposure to varying concentrations of nirogacestat (0.1–1,000 nmol/L). The fold increase in mbBCMA density after 24-hour nirogacestat exposure is shown in **C**. sBCMA concentration after 24-hour exposure to varying concentrations of nirogacestat is shown in **D**. Changes in (**E**) mbBCMA density and (**F**) sBCMA concentration upon exposure to 250 nmol/L nirogacestat for 3, 6, 24, and 48 hours and after washout are shown. All experiments were performed in triplicate, with sBCMA measured in duplicate. ABC, antibody-binding capacity.

Although a spectrum of baseline mbBCMA expression was observed in the six multiple myeloma cell lines before nirogacestat exposure (Fig. 2A), mbBCMA density increased in response to increasing concentrations of nirogacestat, with maximal detectable levels achieved in all cell lines (Fig. 2B). Overall, the mean concentration of nirogacestat required to elicit a EC_50_ was 30.7 (SD, 18.1) nmol/L (Table 1); 250 nmol/L was the lowest nirogacestat concentration that elicited a maximal response across cell lines (Fig. 2B). The maximum increase from baseline in mbBCMA density after nirogacestat treatment ranged from an approximately sixfold increase in H929 cells to an approximate 20-fold increase in U266 and L-363 cells (Fig. 2C). sBCMA concentrations showed an inverse trend to mbBCMA density, decreasing from pre-exposure levels in a concentration-dependent manner in response to nirogacestat (Fig. 2D).

**Table 1:** *In vitro* PD parameters of the nirogacestat effect on mbBCMA in multiple myeloma cell lines

Cell line	EC_50_, nmol/L
MM.1R	10.4
H929	7.9
MOLP-8	41.0
KMS-12-BM	52.6
L-363	41.4
U266	30.9
Mean (SD)	30.7 (18.1)

When the effect of nirogacestat on BCMA dynamics was evaluated over time, the maximum increase in mbBCMA density occurred within 6 hours and was maintained until washout at 48 hours (Fig. 2E). After washout, the kinetics of mbBCMA density reduction were variable. mbBCMA density dropped rapidly toward pre-exposure levels within 3 to 6 hours in four of six cell lines, whereas a slower decline was observed in the remaining two cell lines (MM.1R and H929). In contrast, sBCMA concentrations remained low upon nirogacestat exposure in fresh media over 48 hours and then quickly increased upon nirogacestat washout (Fig. 2F).

To confirm that the effect of nirogacestat on BCMA dynamics is through inhibition of γ-secretase, multiple myeloma cell lines were treated with nirogacestat or two other GSIs at concentrations previously determined to result in a maximal increase in mbBCMA density (250 nmol/L nirogacestat, 100 nmol/L crenigacestat, and 1,000 nmol/L RO4929097). All three GSIs achieved a similar maximal effect in cell lines tested (Supplementary Fig. S2).

### Effect of nirogacestat on BCMA dynamics in healthy volunteers

#### Participants

Participant demographics and baseline characteristics are shown in Supplementary Table S1. Participants were healthy men averaging 28 to 35 years of age and predominantly white (87%, 20/23), with an average body mass index of 22.7 to 25.5 kg/m^2^.

#### PK

**Figure 3:**
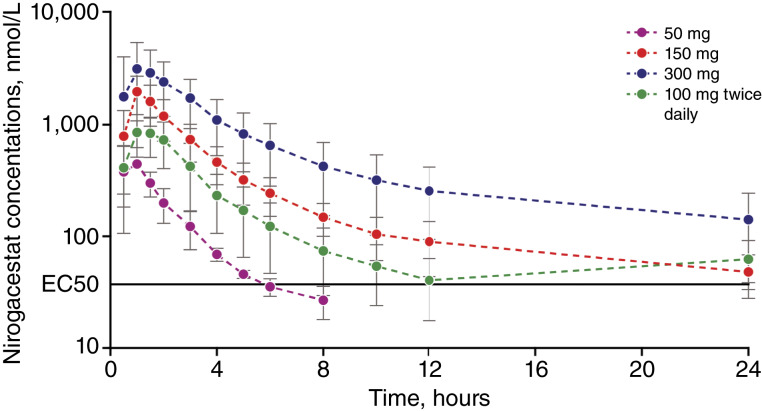
Nirogacestat PK in healthy participants.

After single dose (50, 150, and 300 mg) or repeated dose (100 mg twice daily) nirogacestat administration, serum concentrations of nirogacestat increased rapidly, with a time to peak drug concentration (*T*_max_) of ∼1 hour, and then declined quickly over the first 12 hours (Fig. 3). Nirogacestat exposure tended to increase proportionally to dose. Nirogacestat PK was described by a two-compartment model with linear absorption and linear clearance. A dose effect on bioavailability was noted upon inspection of simulations, so a fit-for-purpose dose effect on bioavailability, as a saturable maximum effect on the bioavailability (*F*_max_) model, was added to the base model. Residual error was low, and inter-interindividual variability was moderate for all three PK parameters. Parameter precision was low, except for the absorption rate (Supplementary Table S2). VPCs and diagnostic plots are shown in Supplementary Figs. S3 and S4.

#### PD

**Figure 4:**
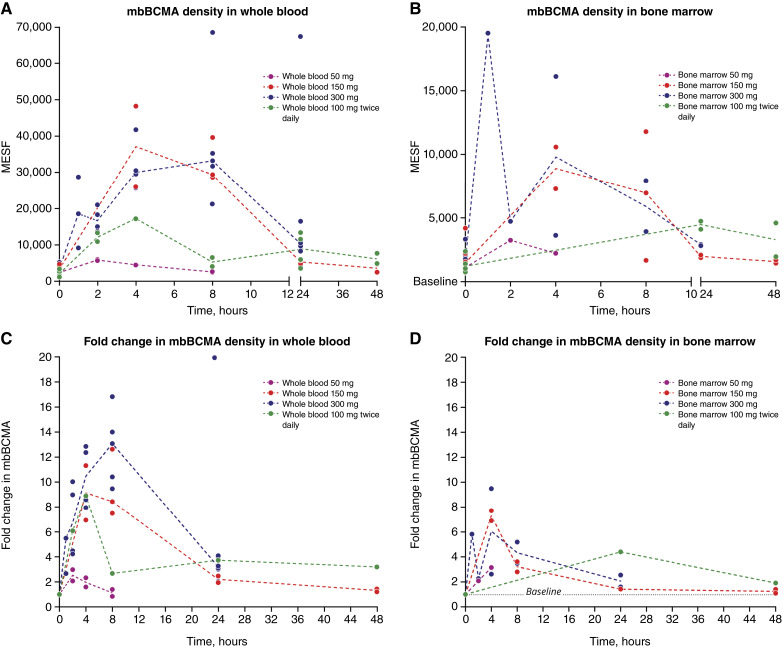
Nirogacestat treatment increases mbBCMA density on PCs from whole blood and bone marrow. mbBCMA density on PCs isolated from (**A**) whole blood or (**B**) bone marrow after single doses of 50, 150, and 300 mg nirogacestat or 2 to 4 100 mg twice daily doses of nirogacestat. Fold change in mbBCMA density on PCs isolated from (**C**) whole blood or (**D**) bone marrow. Data points represent individual sample results, and dashed lines indicate the median for each sample time point; for time points with *n* = 1, the median value is represented by the single value. MESF, molecules of equivalent soluble fluorochrome.

Nirogacestat treatment resulted in rapid (≤1 hour) and robust (9- to 19-fold) increases in mbBCMA density on PCs in both whole blood (Fig. 4A and C) and bone marrow (Fig. 4B and D) after single doses of 50, 150, and 300 mg and repeated doses of 100 mg twice daily. A maximum increase in mbBCMA density occurred within 4 to 8 hours after single doses. A dose-related increase in mbBCMA density was generally observed across the range of administered single doses of nirogacestat (50–300 mg). Increases in mbBCMA density were ∼2-fold greater on PCs isolated from whole blood versus bone marrow. Bone marrow mbBCMA density exhibited greater variability than did whole-blood mbBCMA density, most likely because of the greater complexity of collecting and processing bone marrow samples versus whole blood. The return to baseline of mbBCMA was relatively rapid (24–48 hours after nirogacestat dosing), corresponding with a decline in nirogacestat concentrations (Fig. 4A–D). Following repeated doses of nirogacestat 100 mg twice daily, mbBCMA density generally remained ≥2-fold higher than baseline throughout the dosing interval in both whole blood and bone marrow. Dose-related reductions in sBCMA were observed after treatment with nirogacestat, although the baseline levels were low in this healthy population, and the effect was modest (Supplementary Fig. S5).

**Figure 5:**
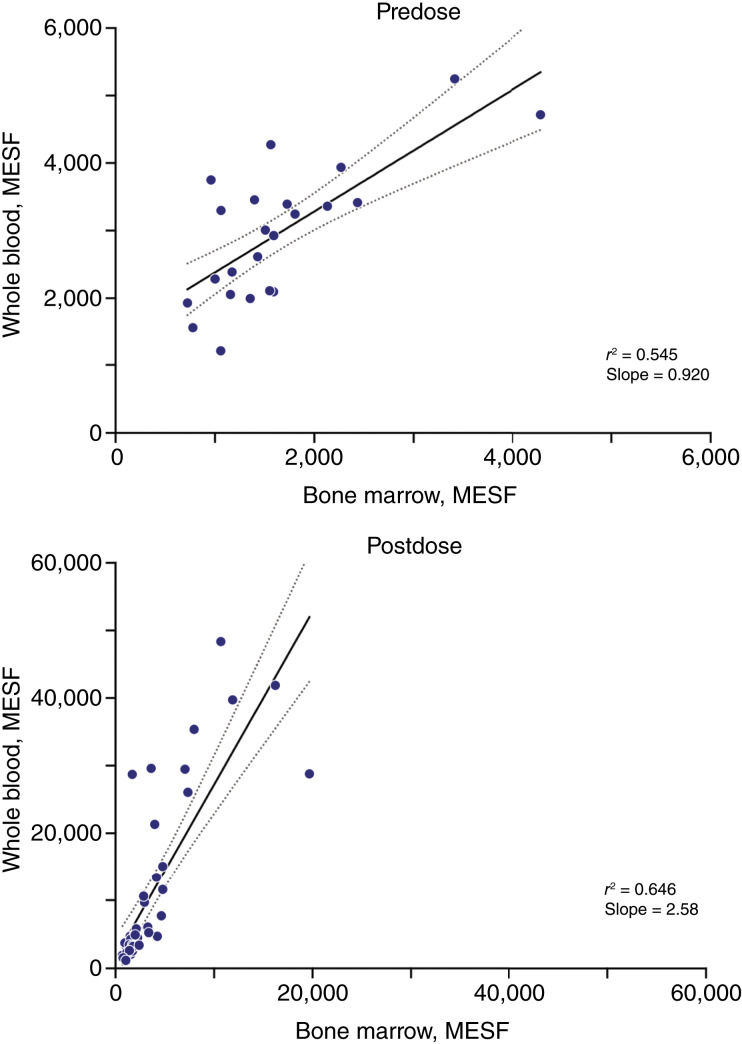
Correlation between BCMA receptor density on PCs isolated from whole-blood and bone marrow samples. MESF, molecules of equivalent soluble fluorochrome; *r*^2^, coefficient of determination of linear regression.

Before and after nirogacestat treatment, BCMA receptor density was correlated between whole blood and bone marrow; however, BCMA response was ∼2.5-fold higher in whole blood versus bone marrow (Fig. 5). Although there was a difference in the magnitude of response in the respective matrices, the close relationship indicates that whole blood may be used as a surrogate for bone marrow when evaluating BCMA receptor density on PCs.

#### Population PKPD model

A sequential PKPD model approach was implemented wherein the final PK model was used to simulate individual concentration-versus-time profiles that were time-matched to mbBCMA values for modeling the PD effect. The PD model of nirogacestat effect on whole-blood mbBCMA included an indirect response model with an effect compartment described by appearance (*k*_in_) and depletion (*k*_out_) of mbBCMA and drug effect as a maximum possible effect (*E*_max_) model with Hill coefficient (γ), in which *k*_in_ was defined as *k*_in_ = *E*0 × *k*_out_ under the steady state assumption (Supplementary Table S3). Observed data were adequately described by the model (prediction–correction VPCs), and the model had good parameter precision (Supplementary Fig. S6). Residual error was high, likely because of too few data points and corresponding high variability (Supplementary Fig. S7; Supplementary Table S3).

#### Safety

In nirogacestat-treated participants, all TEAEs (Supplementary Table S4) were mild in severity. No serious adverse events occurred, and no TEAEs led to study discontinuation.

## Discussion

γ-Secretase inhibition may augment the efficacy of BCMA-targeted therapeutics, and nirogacestat is being evaluated as adjunctive treatment for multiple myeloma in combination with BCMA-directed therapies, including an antibody–drug conjugate and bispecific antibodies. Elucidating the impact of nirogacestat dose on mbBCMA density and the duration of its effect is critical to rational clinical trial design. Here, the discrete impact of nirogacestat on BCMA dynamics was evaluated in multiple myeloma cell lines and in a phase 1 study in healthy volunteers. These studies allowed for the robust characterization of the effects of nirogacestat on multiple myeloma cell lines *in vitro* and on PCs acquired from healthy volunteers *in vivo*, facilitating comparison across investigational platforms. The studies were conducted in the absence of concomitant BCMA-targeting therapeutics to understand the effects of nirogacestat alone, as treatment with antimyeloma agents has been shown to affect BCMA dynamics (8). Nirogacestat does not exhibit direct antimyeloma activity, which necessitated conducting the phase I study in healthy volunteers rather than in patients with multiple myeloma.

The studies yielded consistent results for the kinetics of BCMA dynamics in response to nirogacestat treatment. Nirogacestat increased mbBCMA density rapidly and robustly in a concentration-dependent manner, with maximal increases up to 20-fold occurring within 4 to 8 hours of exposure in multiple myeloma cell lines and in PCs from whole blood or bone marrow of healthy volunteers. Twice daily nirogacestat dosing in healthy volunteers led to a sustained increase in PC mbBCMA density, which generally remained ≥2-fold higher than baseline throughout the dosing interval in both whole blood and bone marrow. Mean EC_50_ observed in multiple myeloma cell lines (30.7 nmol/L) was comparable with the EC_50_ estimated by the PKPD model from the study in healthy volunteers (37.2 nmol/L). After nirogacestat washout (cell lines) or after single-dose peak effects in healthy volunteers, mbBCMA density returned to pre-exposure levels, with a generally quick time course indicative of a short interval to recovered γ-secretase activity. sBCMA showed an opposite trend, decreasing in a concentration-dependent manner in both multiple myeloma cell lines and healthy volunteers in response to nirogacestat, followed by a rapid return toward pre-exposure levels. On the basis of the decline in sBCMA in response to nirogacestat, sBCMA half-life seems to be relatively short. This finding contrasts with a previous study reporting a half-life of 24 to 36 hours based on evaluation of an sBCMA fragment crystallizable fusion protein (21). Because fusion to a fragment crystallizable region is an established method of extending the half-life of proteins (22), this may explain the discrepancy between the present and previous findings. PK findings for nirogacestat, including a short time to maximum concentration and rapid elimination, were consistent with the PD findings, and TEAEs were minimal and mild.

Preclinical and clinical evidence is accumulating that increasing mbBCMA density on PCs through GSI exposure may improve the efficacy of BCMA-targeted therapies in patients with multiple myeloma (8–10). Use of quantitative flow cytometry, as in the present studies, has enabled investigation of this potential mechanism via more sensitive and objective quantification of mbBCMA density over time in the presence and absence of GSIs (15). Quantitative flow cytometric measurement of mbBCMA was also used in the recent Cowan and colleagues (10) phase I study, the first study to evaluate a GSI in combination with a BCMA-targeting therapy in patients with multiple myeloma. In response to GSI exposure in the lead-in of that study, PCs from bone marrow samples showed increases in mbBCMA density of similar magnitude (12.2-fold) to those observed in the present studies in multiple myeloma cell lines and healthy volunteers (up to 20-fold). Results from Cowan and colleagues (10) further suggest that changes observed in mbBCMA density upon GSI exposure may be clinically relevant, as increases in mbBCMA density during the GSI lead-in were associated with significant improvement in progression-free and overall survival. Decreased sBCMA concentration after administration of the GSI/BCMA-targeting therapy combination was also significantly associated with progression-free and overall survival (10). Further studies in larger patient populations will be needed to clarify the relative importance of mbBCMA and sBCMA dynamics to the clinical effects observed.

Elucidating BCMA kinetics in response to nirogacestat is key to guiding potential dosing strategies in multiple myeloma. Nirogacestat administered at 150 mg twice daily has shown efficacy in a phase 3 study of desmoid tumors (17), but a therapeutic dose of nirogacestat as an adjunct to BCMA-directed therapies in multiple myeloma has not yet been established and may be dependent on the BCMA-targeting agent used in combination. Nirogacestat single doses of 50, 150, and 300 mg or multiple doses of 100 mg twice daily achieved peak plasma concentrations well above the EC_50_ values observed in multiple myeloma cell lines and on PCs in healthy volunteers (Fig. 3), suggesting that these doses would be expected to have clinically relevant effects. The rapid BCMA dynamics observed in the present studies indicate that there is no meaningful lag in BCMA response to γ-secretase inhibition with nirogacestat, and therefore, a treatment lead-in period may not be needed. Other potential nirogacestat treatment strategies that should be evaluated include maximizing the BCMA response for a short time and eliciting a sustained BCMA response above a minimum threshold for a defined period. Finally, treatment optimization may involve the initial introduction of a BCMA-targeted therapy to kill off high BCMA-expressing cells, followed by introduction of a GSI, like nirogacestat, to boost mbBCMA on residual multiple myeloma cells.

A practical aspect and strength of the phase I study is the demonstration that whole blood can be used as a surrogate for bone marrow in the determination of mbBCMA density on PCs. Although a PKPD model for bone marrow could not be developed owing to the limited dataset and high sample variability, a correlation was observed between whole-blood and bone marrow mbBCMA densities before and after nirogacestat treatment (*r*^2^ values of 0.545 and 0.646, respectively; see Fig. 5). The increase in mbBCMA density in response to nirogacestat was ∼2.5-fold higher in whole blood–derived PCs versus bone marrow samples, possibly because of differences between the peripheral circulation and the bone marrow microenvironment. Regardless of this difference, results suggest that BCMA dynamics can be monitored in PCs collected from whole blood and reflect biology in the bone marrow, thereby avoiding the need to perform invasive serial bone marrow biopsies.

Limitations of the current analyses are those inherent to *in vitro* and phase I studies. As such, extrapolating results from current studies in multiple myeloma cell lines and healthy volunteers to patients with multiple myeloma may be limited by several factors. PCs constitute <5% of cells in bone marrow of healthy individuals versus ≥10% in patients with active multiple myeloma. Although differences in proportional cell representation may not affect the broader applicability of the present results, potentially altered BCMA dynamics associated with the multiple myeloma disease state remain an important consideration (8, 23). However, we found that the impact of BCMA kinetics on multiple myeloma cell lines was concordant with the impact on PCs from healthy individuals. Similarly, the baseline BCMA expression and the response to the GSI was similar between CD19^+^ cells (utilized for the measurement of BCMA in this study) and CD19^−^ cells obtained from the same patient (Supplementary Fig. S8), suggesting a fidelity between these cell populations irrespective of CD19 status. This represents an important consideration in light of the fact that CD19^−^ cells are more abundant in patients with multiple myeloma. In addition, the eight-color EuroFlow assay is a standardized method for determining minimal residual disease in patients with multiple myeloma and is recommended by the International Myeloma Working Group for assessment of minimal residual disease in patient samples, although notably lacks in the inclusion of a BCMA antibody (24, 25). This standardized assay also includes metrics for the discrimination of normal versus malignant plasma cell populations with a given sample. Although the flow cytometric assay utilized in this study shares similar features to the EuroFlow assay, the nonstandard approach of this assay should be acknowledged and could provide variable results to the standardized panel, particularly in the assessment of patient samples. Furthermore, prior or current exposure to BCMA-targeted therapy can alter BCMA dynamics (8), so the effect of nirogacestat under those circumstances, will need to be evaluated in future studies. Another consideration in generalizing findings from the present studies to patients with multiple myeloma is that nirogacestat was given only in single doses or a small number of repeated doses (four in total) in the present phase 1 study, whereas GSI administration in combination with BCMA-targeted therapy in patients with multiple myeloma would likely involve longer treatment. Finally, the pool of healthy volunteers available was comprised of a relatively homogenous population, and differences in the impact of nirogacestat on PCs from women, older adults, and historically underrepresented populations are possible. Despite these limitations, the consistency of results for BCMA dynamics in response to nirogacestat, observed in multiple myeloma cell lines and healthy volunteers, suggests that these findings may be applicable to patients with multiple myeloma. It should be noted that there was variation in results from the multiple myeloma cell lines, with some cell lines having greater antibody-binding capacity at baseline and concomitantly less increase in mbBCMA density in response to nirogacestat. Such variability in mbBCMA expression may also occur across patients with multiple myeloma or within a single patient owing to cancer cell heterogeneity.

In conclusion, these results provide a pharmacologic profile of the GSI nirogacestat in multiple myeloma cells and humans, showing rapid and robust effects on BCMA dynamics, which support a role for nirogacestat as an adjunctive treatment for multiple myeloma in combination with BCMA-directed therapies.

## Supplementary Material

Supplemental Figure 1 Isolation of BCMA-expressing PCs in whole blood and bone marrow

Supplemental Figure 2 mbBCMA density in MM cells after 24-hour exposure to nirogacestat and other GSIs

Supplemental Figure 3 Visual predictive checks for the nirogacestat dose effect PK model by dose

Supplemental Figure 4 Nirogacestat pharmacokinetic model diagnostic plots

Supplemental Figure 5 Nirogacestat treatment decreases sBCMA concentration

Supplemental Figure 6 Visual predictive checks of the nirogacestat-BCMA PKPD model by dose

Supplemental Figure 7 PKPD model diagnostic plots

Supplemental Figure 8 Comparison of BCMA density (MESF) between CD19- and CD19+ plasma cells in bone marrow (baseline and post-dose samples combined)

Supplemental Table 1 Participant demographics and baseline characteristics

Supplemental Table 2 PK parameter estimates for the nirogacestat dose-effect model

Supplemental Table 3 Parameter estimates for the BCMA PKPD model

Supplemental Table 4 Summary of adverse events
