# Supplementary material for: Kinetics of Nirogacestat-Mediated Increases in B-cell Maturation Antigen on Plasma Cells Inform Therapeutic Combinations in Multiple Myeloma
Source: Cancer Res Commun. 2024 Dec 11;4(12):3114–23. doi: 10.1158/2767-9764.CRC-24-0075 (PMC11632591; doi:10.1158/2767-9764.CRC-24-0075)

Supplemental Figure 1. Isolation of BCMA-expressing PCs in whole blood and bone marrow.

(A) Gating scheme used to isolate BCMA-expressing PCs in whole blood and bone marrow aspirates. Fluorescence was quantified in MESF, which is representative of the number of receptors detected on an individual cell. (B) Distribution of BCMA receptor density on PCs isolated at baseline (blue) and after nirogacestat treatment (red; time points shown as representative examples). BCMA, B-cell maturation antigen; FSC-A, forward scatter area; FSC-H, forward scatter height; FSC-W, forward scatter width; MESF, molecules of equivalent soluble fluorochrome; SSC-A, side scatter area; PC, plasma cell.

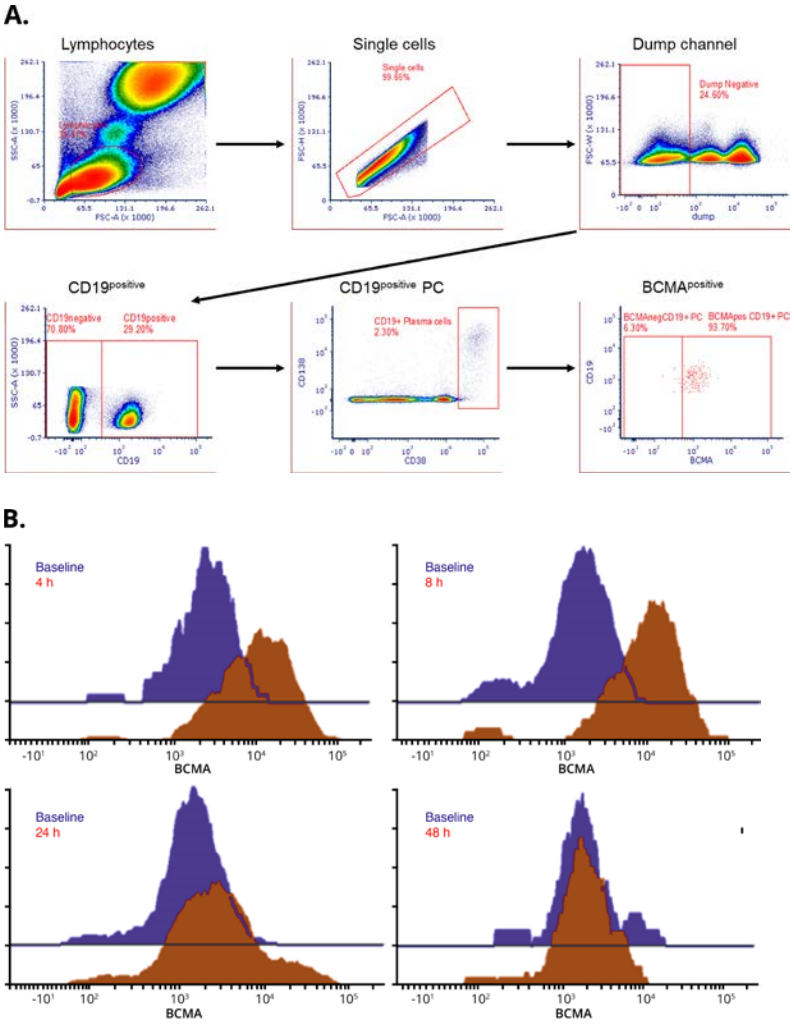

Supplement: Supplemental Figure 1 — Isolation of BCMA-expressing PCs in whole blood and bone marrow [file crc-24-0075_supplemental_figure_1_suppsf1.pdf]
