# Supplementary material for: Kinetics of Nirogacestat-Mediated Increases in B-cell Maturation Antigen on Plasma Cells Inform Therapeutic Combinations in Multiple Myeloma
Source: Cancer Res Commun. 2024 Dec 11;4(12):3114–23. doi: 10.1158/2767-9764.CRC-24-0075 (PMC11632591; doi:10.1158/2767-9764.CRC-24-0075)

Supplemental Figure 2. mbBCMA density in MM cells after 24-hour exposure to nirogacestat and other GSIs. ABC, antibody-binding capacity; BCMA, B-cell maturation antigen; GSI, gamma secretase inhibitor; mbBCMA, membrane-bound BCMA; MM, multiple myeloma.

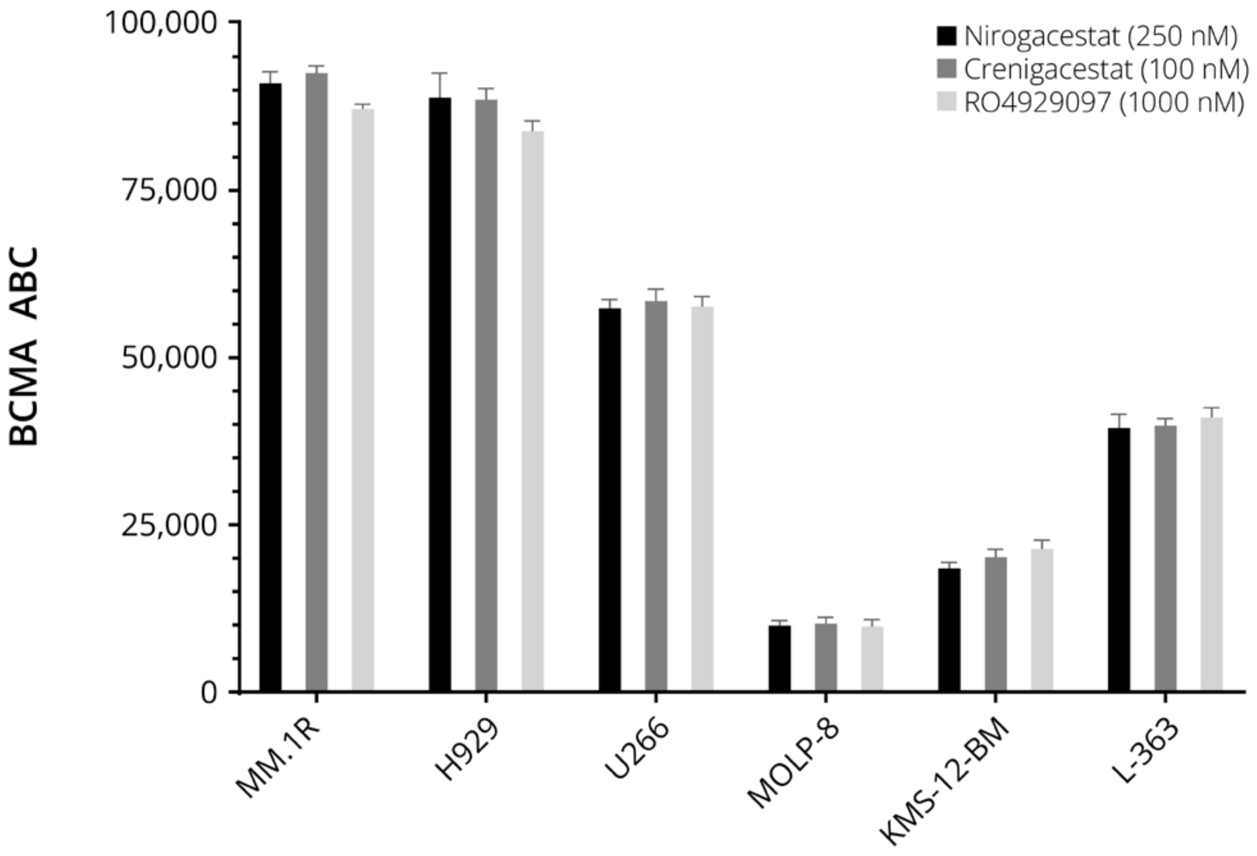

Supplement: Supplemental Figure 2 — mbBCMA density in MM cells after 24-hour exposure to nirogacestat and other GSIs [file crc-24-0075_supplemental_figure_2_suppsf2.pdf]
