# Supplementary material for: Kinetics of Nirogacestat-Mediated Increases in B-cell Maturation Antigen on Plasma Cells Inform Therapeutic Combinations in Multiple Myeloma
Source: Cancer Res Commun. 2024 Dec 11;4(12):3114–23. doi: 10.1158/2767-9764.CRC-24-0075 (PMC11632591; doi:10.1158/2767-9764.CRC-24-0075)

Supplemental Figure 3. Visual predictive checks for the nirogacestat dose effect PK model by dose. BID, twice daily; PK, pharmacokinetic; QD, once daily.

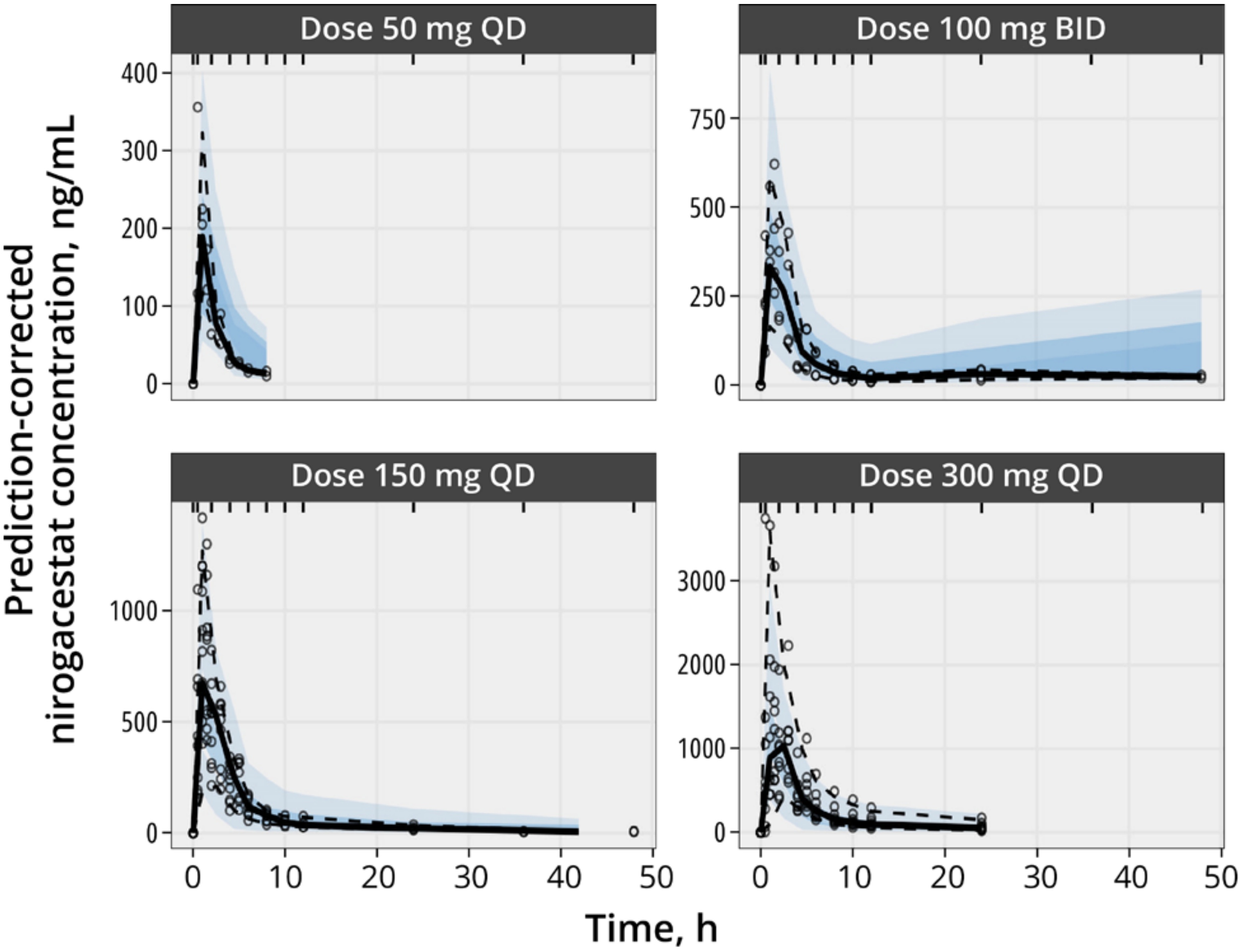

Supplement: Supplemental Figure 3 — Visual predictive checks for the nirogacestat dose effect PK model by dose [file crc-24-0075_supplemental_figure_3_suppsf3.pdf]
