# Supplementary material for: Kinetics of Nirogacestat-Mediated Increases in B-cell Maturation Antigen on Plasma Cells Inform Therapeutic Combinations in Multiple Myeloma
Source: Cancer Res Commun. 2024 Dec 11;4(12):3114–23. doi: 10.1158/2767-9764.CRC-24-0075 (PMC11632591; doi:10.1158/2767-9764.CRC-24-0075)

Supplemental Figure 4. Nirogacestat pharmacokinetic model diagnostic plots. Conc, concentration; CWRES, conditional weighted residuals; DV, dependent variable; IDV, independent variable; IPRED, individual predictions; PRED, population predictions.

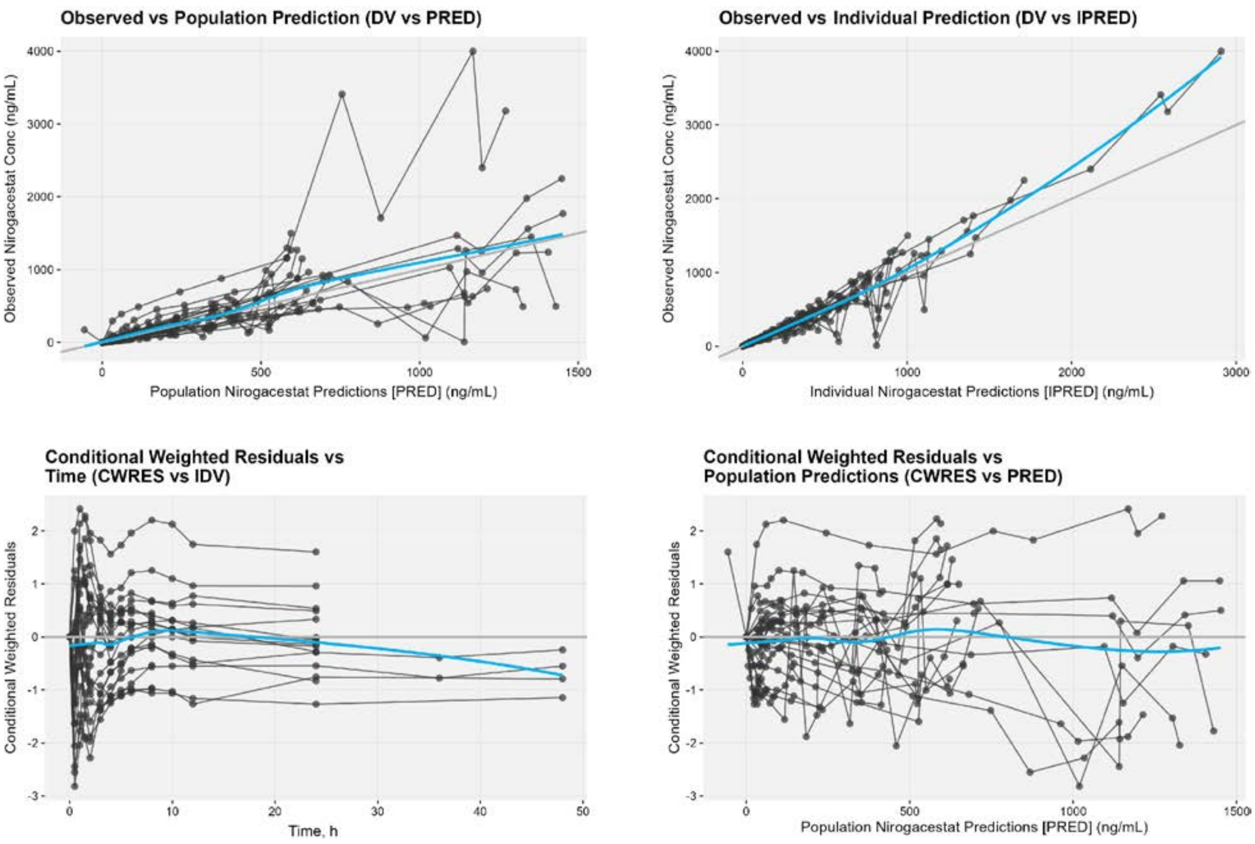

Supplement: Supplemental Figure 4 — Nirogacestat pharmacokinetic model diagnostic plots [file crc-24-0075_supplemental_figure_4_suppsf4.pdf]
