# Supplementary material for: Kinetics of Nirogacestat-Mediated Increases in B-cell Maturation Antigen on Plasma Cells Inform Therapeutic Combinations in Multiple Myeloma
Source: Cancer Res Commun. 2024 Dec 11;4(12):3114–23. doi: 10.1158/2767-9764.CRC-24-0075 (PMC11632591; doi:10.1158/2767-9764.CRC-24-0075)

Supplemental Figure 5. Nirogacestat treatment decreases sBCMA concentration. BCMA, B-cell maturation antigen; sBCMA, soluble BCMA.

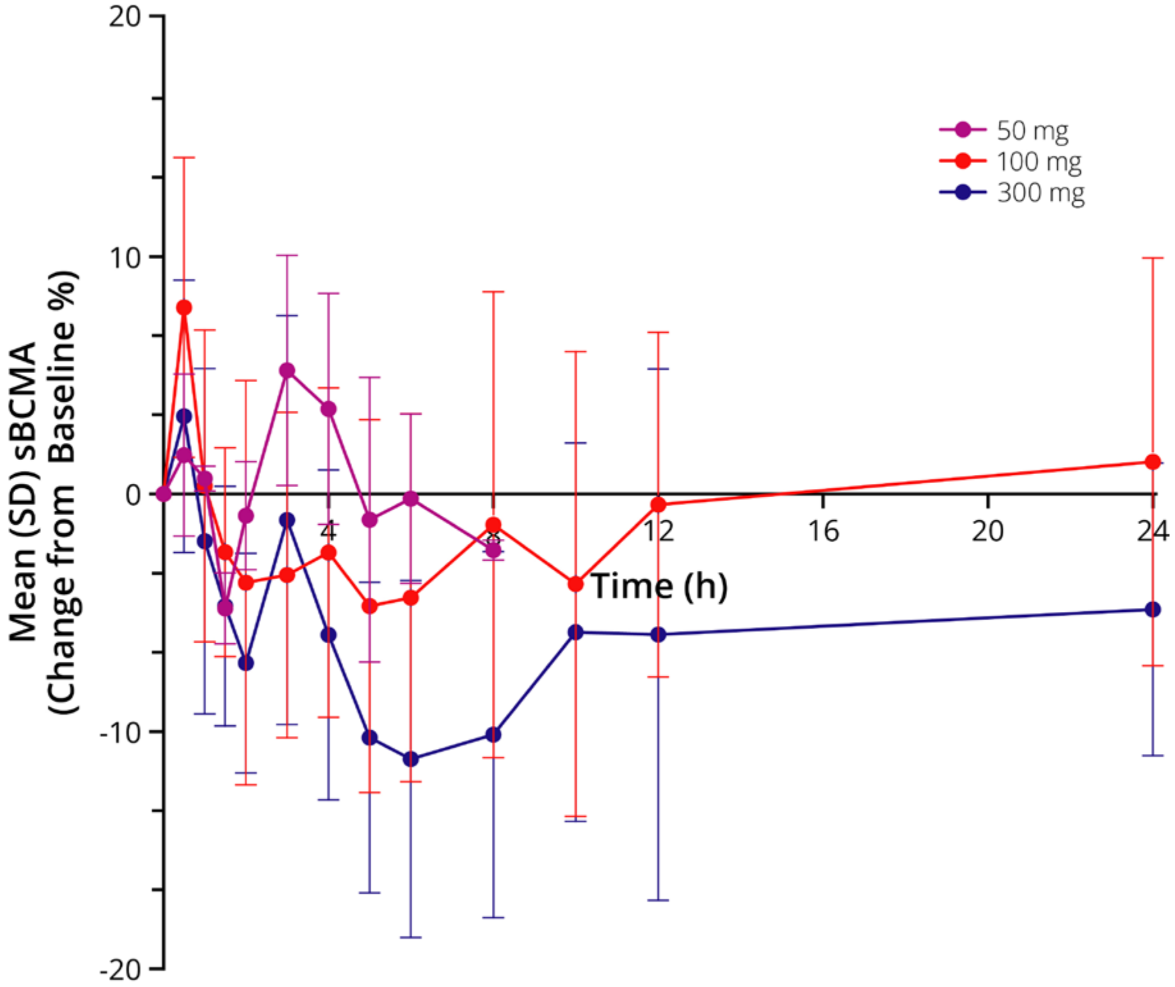

Supplement: Supplemental Figure 5 — Nirogacestat treatment decreases sBCMA concentration [file crc-24-0075_supplemental_figure_5_suppsf5.pdf]
