# Supplementary material for: Kinetics of Nirogacestat-Mediated Increases in B-cell Maturation Antigen on Plasma Cells Inform Therapeutic Combinations in Multiple Myeloma
Source: Cancer Res Commun. 2024 Dec 11;4(12):3114–23. doi: 10.1158/2767-9764.CRC-24-0075 (PMC11632591; doi:10.1158/2767-9764.CRC-24-0075)

**Supplemental Figure 6. Visual predictive checks of the nirogacestat-BCMA PKPD model by dose.** BCMA, B-cell maturation antigen; BID, twice daily; PD, pharmacodynamics; PK, pharmacokinetics; QD, once daily.

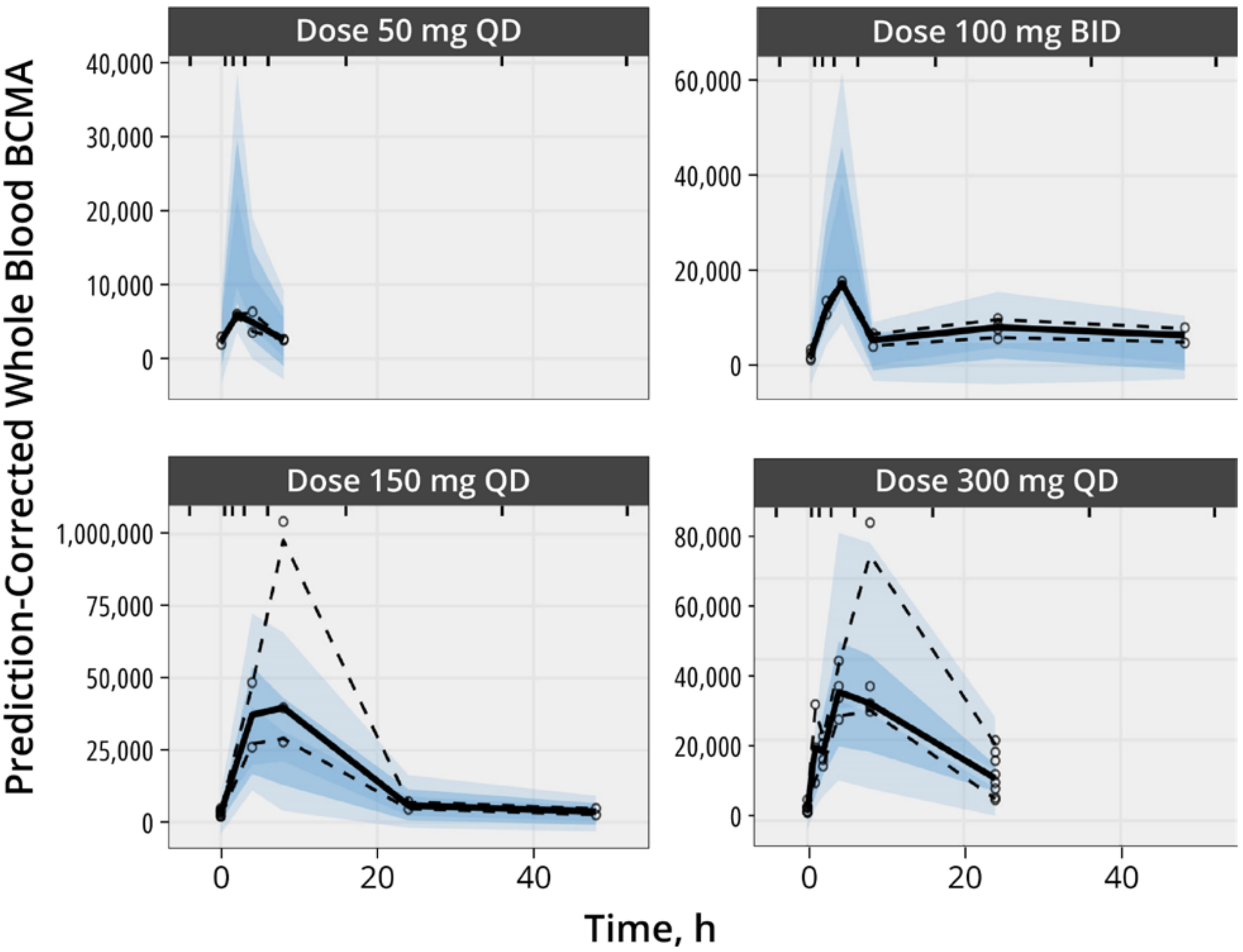

Supplement: Supplemental Figure 6 — Visual predictive checks of the nirogacestat-BCMA PKPD model by dose [file crc-24-0075_supplemental_figure_6_suppsf6.pdf]
