# Supplementary material for: Kinetics of Nirogacestat-Mediated Increases in B-cell Maturation Antigen on Plasma Cells Inform Therapeutic Combinations in Multiple Myeloma
Source: Cancer Res Commun. 2024 Dec 11;4(12):3114–23. doi: 10.1158/2767-9764.CRC-24-0075 (PMC11632591; doi:10.1158/2767-9764.CRC-24-0075)

Supplemental Figure 8. Comparison of BCMA density (MESF) between CD19- and CD19+ plasma cells in bone marrow (baseline and post-dose samples combined).

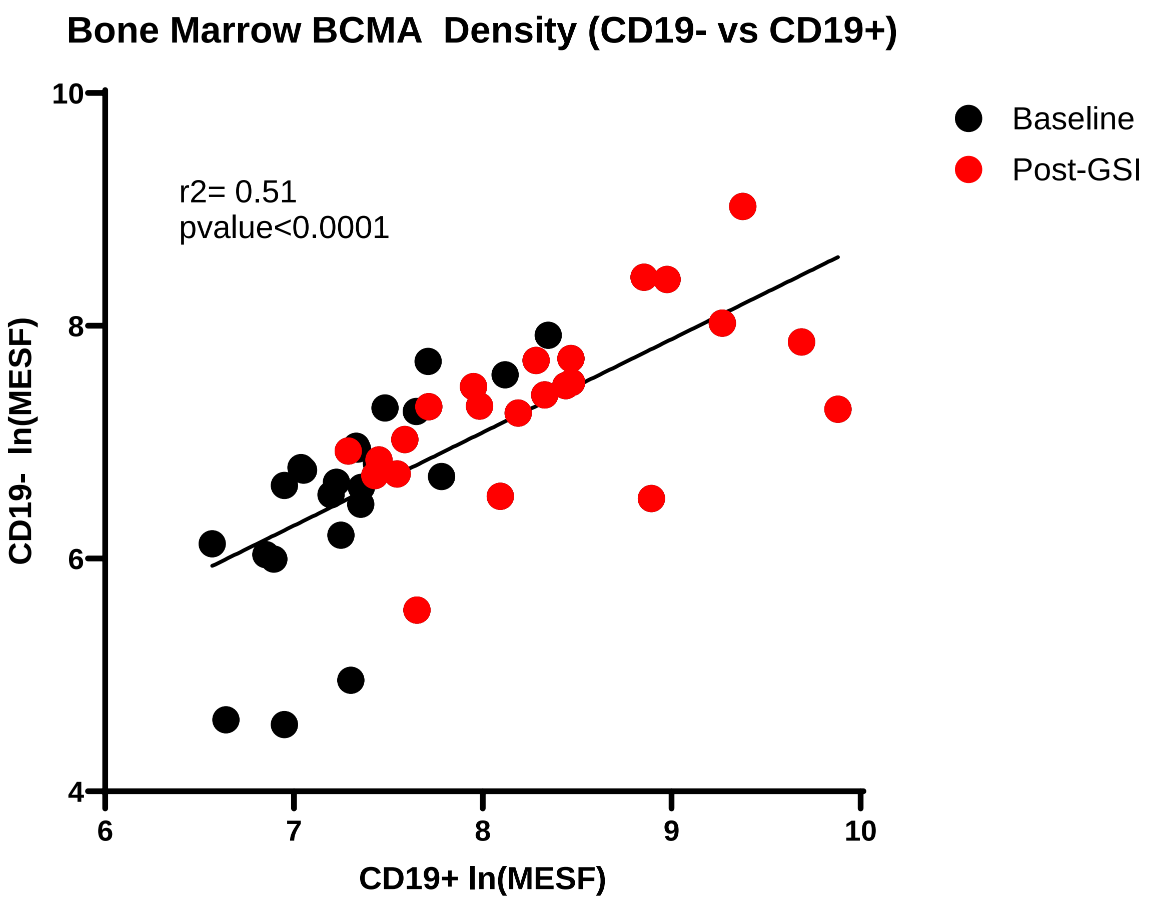

Supplement: Supplemental Figure 8 — Comparison of BCMA density (MESF) between CD19- and CD19+ plasma cells in bone marrow (baseline and post-dose samples combined) [file crc-24-0075_supplemental_figure_8_suppsf8.pdf]
