# Supplementary material for: Kinetics of Nirogacestat-Mediated Increases in B-cell Maturation Antigen on Plasma Cells Inform Therapeutic Combinations in Multiple Myeloma
Source: Cancer Res Commun. 2024 Dec 11;4(12):3114–23. doi: 10.1158/2767-9764.CRC-24-0075 (PMC11632591; doi:10.1158/2767-9764.CRC-24-0075)
Supplement: Supplemental Table 1 — Participant demographics and baseline characteristics [file crc-24-0075_supplemental_table_1_suppst1.pdf]

Supplemental Table 1. Participant demographics and baseline characteristics.

Characteristic	Nirogacestat 50 mg (n=2)	Nirogacestat 150 mg (n=9)	Nirogacestat 300 mg (n=8)	Nirogacestat 100 mg BID (n=4)	
				2 doses (n=2)	4 doses (n=2)
**Male, n (%)**	2 (100)	9 (100)	8 (100)	2 (100)	2 (100)
**Age, mean (range), y**	29 (27-31)	32 (19-55)	35 (25-53)	28 (27-28)	30 (27-33)
**Race, n (%)**					
White	0	8 (88.9)	8 (100)	2 (100)	2 (100)
American Indian or Alaska Native	1 (50)	0	0	0	0
Multiple	1 (50)	1 (11.1)	0	0	0
**Ethnicity, not Hispanic/Latino, n (%)**	1 (50)	9 (100)	8 (100)	2 (100)	2 (100)
**BMI, mean (SD), kg/m^2**	25.5 (0.2)	24.7 (1.9)	24.8 (2.3)	23.3 (3.2)	22. 7 (2.1)

BID, twice daily; BMI, body mass index.
