# Supplementary material for: Kinetics of Nirogacestat-Mediated Increases in B-cell Maturation Antigen on Plasma Cells Inform Therapeutic Combinations in Multiple Myeloma
Source: Cancer Res Commun. 2024 Dec 11;4(12):3114–23. doi: 10.1158/2767-9764.CRC-24-0075 (PMC11632591; doi:10.1158/2767-9764.CRC-24-0075)
Supplement: Supplemental Table 2 — PK parameter estimates for the nirogacestat dose-effect model [file crc-24-0075_supplemental_table_2_suppst2.pdf]

Supplemental Table 2. PK parameter estimates for the nirogacestat dose-effect model.

Parameter	Estimate	%RSE	IIV
**CL, L/h (95% CI)**	44.2 (36.1, 54.1)	0.962	56.5
**V, L (95% CI)**	44 (36, 54)	0.946	49
**Ka, h^{-1} (95% CI)**	1.49 (1.09, 2.02)	40	44.8
**Q, L/h (95% CI)**	28.2 (22.8, 34.9)	1.06	NA
**V2, L (95% CI)**	352 (291, 426)	0.762	NA
**F1, fraction**	0.171 fixed	NA	NA
**F_{max}**	2 fixed	NA	NA
**D_{50}, mg**	150 fixed	NA	NA
**Proportional error, %**	24.7	NA	NA

CL, clearance; D_{50}, dose that exhibits 50% of maximum effect on bioavailability; F1, bioavailability; F_{max}, maximum effect on bioavailability; IIV, interindividual variability; Ka, absorption rate; NA, not applicable; PK, pharmacokinetics; Q, intercompartmental distribution rate; RSE, relative standard error; V, volume of distribution; V2, volume of distribution of second compartment.
