# Supplementary material for: Kinetics of Nirogacestat-Mediated Increases in B-cell Maturation Antigen on Plasma Cells Inform Therapeutic Combinations in Multiple Myeloma
Source: Cancer Res Commun. 2024 Dec 11;4(12):3114–23. doi: 10.1158/2767-9764.CRC-24-0075 (PMC11632591; doi:10.1158/2767-9764.CRC-24-0075)
Supplement: Supplemental Table 3 — Parameter estimates for the BCMA PKPD model [file crc-24-0075_supplemental_table_3_suppst3.pdf]

Supplemental Table 3. Parameter estimates for the BCMA PKPD model.

Parameter	Estimate	%RSE	IIV
**EC_{50}, nM (95% CI)**	37.2 (26, 53.3)	5.06	NA
**E_0, MESF (95% CI)**	2170 (1630, 2890)	1.9	58.9
**E_{max}**	0.92 fixed	NA	1
**k_{out}, h^{-1}**	4.16 fixed	NA	NA
**Gamma**	2 fixed	NA	NA
**Residual additive error, MESF**	2940	NA	NA

Note: k_{in} (conceptually the increase of membrane-bound BCMA) is defined in the model as $E0*k_{out}$

BCMA, B-cell maturation antigen; EC_{50}, concentration of nirogacestat required to elicit a 50% maximal response; E_0, baseline MESF; E_{max}, maximum MESF; gamma, Hill slope; IIV, interindividual variability; k_{out}, receptor turnover rate; MESF, molecules of equivalent soluble fluorochrome; NA, not applicable; PD, pharmacodynamics; PK, pharmacokinetics; RSE, relative standard error.
