# Supplementary material for: Kinetics of Nirogacestat-Mediated Increases in B-cell Maturation Antigen on Plasma Cells Inform Therapeutic Combinations in Multiple Myeloma
Source: Cancer Res Commun. 2024 Dec 11;4(12):3114–23. doi: 10.1158/2767-9764.CRC-24-0075 (PMC11632591; doi:10.1158/2767-9764.CRC-24-0075)
Supplement: Supplemental Table 4 — Summary of adverse events [file crc-24-0075_supplemental_table_4_suppst4.pdf]

Supplemental Table 4. Summary of adverse events.

Category, n (%)	Nirogacestat 50 mg (n=2)	Nirogacestat 150 mg (n=9)	Nirogacestat 300 mg (n=8)	Nirogacestat 100 mg BID (n=4)	
				2 doses (n=2)	4 doses (n=2)
**TEAEs[a]**	0	4 (44)	2 (25)	2 (100)	1 (50)
Related	0	2 (22)	0	0	1 (50)
**SAEs**	0	0	0	0	0
**TEAEs leading to withdrawal**	0	0	0	0	0
**All adverse events[b]**					
Flatulence	0	0	0	2 (100)	1 (50)
Frequent bowel movements	0	1 (11)	0	0	0
Nausea	0	1 (11)[c]	0	0	0
Procedural pain	0	1 (11)	0	0	0
Catheter site pain	0	0	1 (13)	0	0
Fatigue	0	1 (11)[c]	0	0	0
Somnolence	0	0	0	1 (50)	1 (50)[c]

Musculoskeletal stiffness	0	0	1 (13)	0	0
Blister	0	1 (11)	0	0	0

BID, twice daily; SAE, serious adverse event; TEAE, treatment-emergent adverse event.

[a]All TEAEs were deemed mild in severity.

[b]Most TEAEs were unrelated to treatment.

[c]TEAEs deemed related to treatment.
